# Optic nerve sheath diameter in severe preeclampsia with neurologic features versus controls

**DOI:** 10.1186/s12884-022-04548-8

**Published:** 2022-03-19

**Authors:** Mary E. Sterrett, Brittany Austin, Ryan M. Barnes, Eugene Y. Chang

**Affiliations:** 1 Department of Obstetrics and Gynecology, Medical University of South Carolina, Charleston, SC USA; 2 Department of Obstetrics and Gynecology, University of Washington USA, Office 356460, 6th floor, 1959 NE Pacific Street, Seattle, WA 98195 USA; 3 Department of Emergency Medicine, Medical University of South Carolina, Charleston, SC USA

**Keywords:** Pregnancy, Preeclampsia, Ultrasonography, Point-of-care systems*, Optic Nerve

## Abstract

**Background:**

Optic nerve sheath diameters (ONSD) have been validated as an accurate screening tool to detect elevated intracranial pressure in hypertensive encephalopathy. The neurologic manifestations of preeclampsia and/or eclampsia mimic those of hypertensive encephalopathy. This study was performed to assess the incidence of elevated optic nerve sheath diameters of patients with severe preeclampsia and neurologic criteria compared to non-preeclamptic patients. The secondary objective was to determine baseline optic nerve sheath diameters in patients with severe preeclampsia without neurologic criteria and preeclampsia without severe features.

**Methods:**

Single site cohort study including 62 pregnant women 18 years or older and 20 weeks or further gestation. Patients with preeclampsia without severe features, preeclampsia with severe features by non-neurologic criteria, preeclampsia with severe features with neurologic criteria, and patients without preeclampsia were enrolled via convenience sampling. One blinded reviewer measured sheath diameters; baseline demographics and pregnancy data were collected by chart review. Statistical analysis was completed with STATA/IC 16. Categorical variables were compared by the χ^2 ^test. Continuous variables were presented as mean ± standard deviation, and discrete variables were presented as medians and compared by Kruskal–Wallis testing. Normality was confirmed by Shapiro–Wilk testing. Linear and logistic regression were used to test the association between the preeclampsia groups and optic nerve sheath diameters. Models were presented as unadjusted and adjusted for BMI, gestation, hypertension, diabetes, parity, and gravidity.

**Results:**

The incidence of optic nerve sheath diameters > 5.8 mm was 43.8% in the severe preeclampsia with neurologic features cohort, and 42.1% in the control cohort, with a relative risk of 1.04. Patients with severe preeclampsia without neurologic features had sheath diameters of 5.75 mm ± 1.09 mm; non-severe preeclampsia patients had sheath diameters of 5.54 mm ± 1.26 mm.

**Conclusions:**

We did not find a significant elevated optic nerve sheath diameter relative risk between severe preeclampsia patients with neurologic features and non-preeclampsia control patients. This is the first study to assess a North American population utilizing ACOG criteria for severe and non-severe preeclampsia, with severe cohorts additionally stratified by neurologic criteria.

## Background

Preeclampsia (PE) is an obstetrical complication characterized by new-onset hypertension or super-imposed hypertension with or without one systemic condition including proteinuria, hepatic dysfunction, neurologic symptoms, renal insufficiency, pulmonary edema, or thrombocytopenia during the second half of pregnancy.

The clinical manifestations of severe preeclampsia can be confusing for the clinician, as several subjective symptoms such as headache, epigastric pain, or right upper quadrant pain can be benign findings in pregnancy. Although hypertension in pregnancy is acknowledged to be a continuum of risk, and early delivery is indicated for many women, identifying an objective measure of lower risk could be useful in stratifying the need for early term delivery to avoid complications of premature delivery, and subsequent maternal management.

The clinical neurologic manifestations of preeclampsia and/or eclampsia have multiple theories about pathogenesis. One theory is that hyper perfusion of the brain due to decreased cerebrovascular resistance leads to vasogenic edema, similar to hypertensive encephalopathy [1, 2]. Hypertensive encephalopathy is hypothesized to be an overperfusion injury causing disordered cerebral autoregulation and subsequent globally decreased cerebral blood flow with extravasation of fluid into the cerebral parenchyma [3, 4]. Similarly, the results of noninvasive studies of cerebral blood flow and resistance suggest that vascular barotrauma and loss of cerebral vascular autoregulation contribute to the cerebral vascular pathology in preeclampsia [5–7]. Radiologically, this can be visualized via computed tomography and magnetic resonance imaging demonstrating cerebral edema in some women with severe preeclampsia or eclampsia [1].

Cerebral edema is believed to cause this increase in intracranial pressure. The subarachnoid spaces surrounding the optic nerve communicate with the intracranial cavity, and changes in cerebrospinal fluid pressure are transmitted along the distensible optic nerve sheath to increase sheath diameter [8, 9]. Two recent meta-analyses suggest that optic nerve sheath diameters can be used as surrogate markers for elevated intracranial pressure, but cite a range of cut-off from 4.8 to 6.3 mm based on the multiple studies which were included [10, 11]. The wide range in potentially significant ONSD measurements raises the question of what an expected ONSD in an obstetric population is. In a comparison of ocular ultrasonography with gold standard measures of intracranial pressure (ICP) via invasive devices such as intraventricular catheters, values of ONSD above 5.8 mm were shown to be associated with a 95% risk of raised ICP (i.e. more than 20 mmHg) [12], which is why we chose to use this threshold, but the optimal ONSD cuff off value for raised ICP is unknown.

To date, there have been four studies evaluating ONSD in preeclampsia and eclampsia. Dubost et al. evaluated a total of 51 women: 26 preeclamptic patients, of which 13 had severe preeclampsia, and 13 had preeclampsia without severe features. A self-reported weakness of the Dubost study was the small percentage of severe preeclampsia patients with neurologic symptoms, limiting the ability to determine a relationship with ONSD enlargement [13]. Only 8 women with preeclampsia had headaches, which included both severe (*n* = 7) and non-severe preeclamptic (*n* = 1) patients. The sample included preeclampsia patients predominantly diagnosed (54%) with severe features by renal dysfunction, which is reported only in approximately 1% of women with severe preeclampsia [14]. They found that approximately 19% of severe preeclamptic patients had ONSD values indicating intracranial pressures above 20 mmHg [13].

Simenc et al. assessed 30 severe preeclampsia patients and compared their ONSD and optic disc height (ODH) measurements to control patients [15]. They did not assess preeclamptics without severe features or differentiate patients by subjective or objective features of severe preeclampsia. They found 43% of patients with severe preeclampsia had ONSD measurements > 5.8 mm and 77% with an ODH ≥ 1 mm, compatible with intracranial hypertension [15]. Ortner et al. evaluated point of care ultrasound (POCUS) in 95 severe preeclampsia patients, which included the incidence of elevated ONSD in this population [16]. However, their primary outcome was the relationship of albumin to POCUS for pulmonary edema or elevated ONSD above 5.8 mm [16]. Singh and Bhatia evaluated 75 pregnant patients, in cohorts of 25 patients of severe preeclampsia, eclampsia, and control groups. They found significant differences between the ONSD measurements in the three cohorts with 44% of preeclampsia patients and 66% of eclampsia patients demonstrating elevations of ONSD values ≥ 5.7 mm [17]. Of note, they had included patients with IUGR for diagnosis of severe preeclampsia, and excluded those with blurred vision, moderate-to-severe renal or hepatic dysfunction or coagulopathy [17]. Exclusion of patients with laboratory criteria for severe preeclampsia would remove 7.3% of patients with preeclampsia, especially as the frequency of abnormal laboratory values in women with pregnancy-associated hypertension increases with disease severity [18]. Evaluating ONSDs in a North American population with severe preeclampsia by ACOG criteria is a necessary step to begin assessing utility of this point of care technique to our obstetric population.

This aim of this study was to estimate the incidence of elevated ONSD in severe preeclampsia patients with neurologic features compared to non preeclamptic patients. The secondary objective was to determine baseline optic nerve sheath diameters in patients with severe preeclampsia with and without neurologic criteria and preeclampsia without severe features. We anticipated correlation of clinical features of severe preeclampsia to elevated ONSD representing vasogenic edema.

## Methods

### Design

This was a prospective study including four pregnant cohorts: patients without preeclampsia (“controls”); patients with PE without severe features; patients with severe PE (sPE) but no neurologic findings, and patients with sPE and neurologic findings. Recruitment began in November 2018 and continued until January 2020, when access to the study-specific ultrasound was expected to cease. Participants were recruited from the inpatient or antepartum unit of Labor and Delivery at a major university hospital in a southern state.

### Sample

Patients were enrolled by convenience sampling. Inclusion criteria included women with a singleton, nonanomalous pregnancy, participants > 20 weeks gestation, and 18 years old or older at the time of recruitment. Exclusion criteria included patients with preexisting eye conditions or ocular surgeries as well as any woman with pseudotumor, intracranial hypertension, seizure disorder, or known intracranial pathology.

Preeclampsia by ACOG criteria is traditionally defined by a patient having two elevated blood pressures of 140/90 four hours apart with a urine protein to creatinine ratio of 0.3 or higher. If the patient did not meet urinary protein criteria, they could also be diagnosed by severe features, including elevated liver enzymes to twice the upper limit of normal, elevated creatinine of greater than 1.1 mg/dL, pulmonary edema, new-onset headache unresponsive to acetaminophen, visual disturbances, severe persistent right upper quadrant or epigastric pain, a platelet count less than 100 × 109 /L, or severe range blood pressures of 160 mm Hg or higher, diastolic blood pressure of 110 mm Hg or higher.

We powered this study by assuming a relative risk of 8.6 [15], expected incidence of elevated ONSD in non-preeclamptic patients of 5% [19], confidence level of 0.95, and a power of 0.8. This required 16 patients in each cohort.

### Data collection

Patients were interviewed at the time of enrollment and asked their age, ethnicity, and history of intracranial pathology. BMI, blood pressures, gravidity and parity, and gestational age were recorded from their medical record. Subjects were asked about symptoms of severe preeclampsia, including persistent headaches, visual scotomata or changes, right upper quadrant or epigastric pain, and shortness of breath that would indicate pulmonary edema. Informed consent was obtained in writing with an IRB-approved form.

Subjects were placed in a semi-recumbent position with the upper part of the body and head at 30–45 degrees from the vertical position for three minutes or less to obtain ultrasound data. A thick layer of water-soluble gel was applied to the orbital fossa with the eye closed, in accordance with prior published protocols [20–22]. A single ultrasonographer (M.S.) previously trained in obtaining optic nerve sheath images by R.B. performed all ultrasound scans. A 13-6 MHz linear probe (L25/13–6 MHz, of Fujifilm SonoSite, Bothell, WA) was utilized [23, 24]. The transducer was gently placed over the fossa to visualize the globe and surrounding structures [25]. Multiple still images of each eye were saved for subsequent review and measurement.

An attending emergency room physician (R.B.) trained in ocular ultrasound performed a blinded assessment of de-identified images to obtain ONSD measurements. This was done in a post hoc fashion directly on the ultrasound machine. ONSD was measured 3 mm behind the globe using an electronic caliper at an axis perpendicular to the optic nerve. Two ONSD measurements were taken in each eye, and the mean of the measurements was used. If the two measurements could not be taken, then one data point was used instead of a mean. A maximum of four values were taken, and the mean values for each eye, or one value if only one clear image was obtained, was stored for data analysis.

### Statistical analysis

Statistical analysis was completed with STATA/IC 16 (StataCorp. 2019. Stata Statistical Software: Release 16. College Station, TX: StataCorp LLC). We powered our study using the elevated ONSD incidence of 43% from Simenc et al. [15], and an anticipated baseline elevated ONSD rate of 5% [19]. To obtain a power of 95%, using an alpha level of 0.05, we required 16 patients in our severe preeclampsia cohort with neurologic features and 16 in our non-preeclamptic cohort to assess the primary outcome of significant difference in ONSD means between the two groups. Our secondary outcome was to assess if there was a significant difference between any of the cohorts’ ONSD means.

Categorical variables of chronic hypertension (cHTN) and preexisting diabetes (DM) were presented as total numbers and compared by the χ^2 ^test. Continuous variables of gestational age and BMI were presented as mean ± standard deviation, and discrete variables of gravidity and parity were presented as medians and compared by Kruskal–Wallis testing. The normality of the ONSD data was confirmed by Shapiro–Wilk testing.

Linear and logistic regression was used to test the association between the preeclampsia groups and ONSD. Models were presented as unadjusted and adjusted for BMI, gestation, hypertension, diabetes, parity, and gravidity. The incidence of elevated ONSD in each group was calculated, and the relative risk of elevated ONSD was assessed. Statistical analysis was completed with STATA/IC 16 (StataCorp. 2019. Stata Statistical Software: Release 16. College Station, TX: StataCorp LLC.

## Results

A total of 62 patients were enrolled. Demographic data is included in Table 1: Patient Demographics. We adjusted for BMI, gestation, cHTN, DM, parity, & gravidity and found no significant difference during our regression analysis.

**Table 1:** Patient demographics

	Total *n* = 62	sPE with Neurologic Features *n* = 16	sPE without Neurologic Features *n* = 19	Preeclampsia *n* = 8	Controls *n* = 19	*P*-value
BMI (kg/m^2^)	34.24 ± 8.9	32.40 ± 8.01	36.59 ± 9.24	34.54 ± 11.20	33.26 ± 8.90	*p* = 0.53^‡^
Gestation (weeks)	34.25	31.4 ± 3 .11	32.57 ± 4.50	35.25 ± 3.66	37.8 ± 3.91	*p* = 0.12^‡^
Chronic HTN	11	4 (25%)	5 (26.3%)	0	2 (10.5%)	*p* = 0.27^†^
Preexisting DM	9	1 (6.3%)	4 (21.1%)	1 (12.5%)	3 (15.8%)	*p* = 0.44^†^
Gravidity	2	1.5	2	1	2	*p* = 0.30*
Parity	0	0	1	0	0.5	*p* = 0.27*

*Legend:sPE* Severe Preeclampsia, *HTN* Hypertension, *DM* Diabetes Mellitus

^†^Compared by χ2

^‡^Compared by one way ANOVA

^*^Compared by Kruskal–Wallis

For the purposes of our study, an elevated ONSD was defined as a measurement > 5.8 mm. The mean ONSD with confidence intervals and incidence of elevated ONSD was calculated for each cohort, and unadjusted and adjusted results are shown in Table 2. The incidence of elevated ONSD in the severe preeclampsia cohort with neurologic features was 44%, and the incidence in the non-preeclampsia control group was 44%. The relative risk for having an elevated ONSD with severe preeclampsia with neurologic features was 1.04. We also calculated the odds ratio, which resulted in an OR of 0.97 for having an elevated ONSD for the severe preeclampsia cohort with neurologic features. For example, we would expect, on average, that those in the sPE with neurologic features cohort to have an ONSD of 0.26 mm lower than the patients without preeclampsia (control) cohort (Table 2).

**Table 2:** Association of preeclampsia cohorts with ONSD

	**n**	**Outcome = Mean (SD)**	**Continuous ONSD**	***P***-**value**	Adjusted * *p*-value	***P*-value**
			**Unadjusted β (95% CI)**			
sPE with Neuro	16	5.67 (0.78)	-0.26 (-0.92, 0.40)	0.440	-0.37 (-1.19, 0.45)	0.367
sPE without Neuro	19	5.75 (1.08)	-0.17 (-0.80, 0.46)	0.593	-0.25 (-1.01, 0.51)	0.506
Mild PE	9	5.51 (1.18)	'-0.41 (-1.20, 0.37)	0.299	'-0.58 (-1.43, 0.28)	0.184
Normal	18	5.92 (0.84)	0 (ref)		0 (ref)	
	**n**	**Outcome = *n* > 5.80 (%)**	**Dichotomous ONSD**	** > 5.8 mm**		
			**Unadjusted**	***P***-**value**	Adjusted *	***P***-**value**
			**OR (95% CI)**		**OR (95% CI)**	
sPE with Neuro	16	7 (44%)	0.97 (0.25, 3.78)	0.968	0.79 (0.14, 4.31)	0.783
sPE without Neuro	19	10 (53%)	1.39 (0.38, 5.07)	0.619	1.24 (0.27, 5.80)	0.785
Mild PE	9	1 (11%)	0.16 (0.02, 1.52)	0.110	0.13 (0.01, 1.35)	0.087
Normal	18	8 (44%)	1.00 (ref)		1.00 (ref)	

^*^adjusted for BMI, gestation, cHTN, DM, parity, & gravidity

**Fig. 1:**
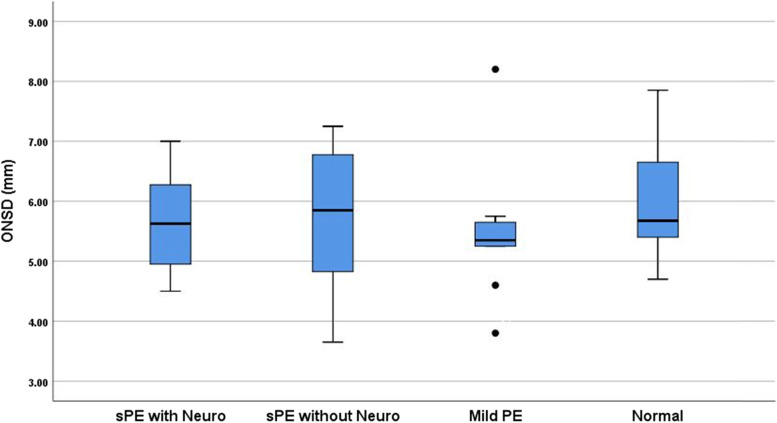
Distribution of Optic Nerve Sheath Diameters. Legend: ONSD: Optic nerve sheath diameters (mm). sPE with Neuro: Severe preeclampsia patients with neurologic features. sPE without Neuro: Severe preeclampsia patients without neurologic featues. Mild PE: Preeclampsia patients without severe features. Normal: Control patients

There was not a statistically significant difference in ONSD between the four groups, χ2(3) = 3.56, *p* = 0.313. The control group had the largest diameters without significant difference in ONSD means between cohorts. Severe preeclampsia with neurologic features had overlap in ONSD measurements with all three cohorts (Fig. 1).

## Discussion

The ONSD has been validated as an accurate screening tool to detect elevated intracranial pressure in patients with idiopathic intracranial hypertension, traumatic brain injury, and spontaneous intracranial hemorrhage [26, 27].

This study’s lack of a significant increase in ONSD measurements in the severe preeclampsia cohorts was an unexpected outcome, as Simenc et al.’s study noted a significant increase in the percentage of ONSD measurements > 5.8 mm in severe preeclampsia groups compared to controls [15]. Limitations of the size of the cohorts could be causing a type II error regarding this outcome.

Belfort et al. have studied the use of transcranial Doppler and shown that a majority of women with preeclampsia have altered cerebral perfusion pressure (CPP) [28] although the etiology may be different between mild and severe disease states. Specifically, women with mild preeclampsia had reduced CPP compared to normal pregnant women, compared to an increase in CPP for women with severe preeclampsia [28]. The authors’ theory was that in preeclampsia without severe features, the predominant abnormality was hypoperfusion (suggestive of vasospasm and ischemia) [2], and in severe preeclampsia women had hyper perfusion (suggestive of hypertensive encephalopathy) [2, 28]. Their findings track with our results of women with preeclampsia without severe features having the overall lowest mean ONSD of 5.54 mm, although the differences in ONSD measurements between our cohorts was not significant. The preeclampsia without severe features cohort had only one patient with an ONSD of > 5.8 mm, giving an incidence of 12.5% for an elevated ONSD measurement consistent with elevated ICP.

### Limitations and strengths

This is the first study to assess a North American population utilizing ACOG criteria for non-severe and severe preeclampsia stratified by neurologic criteria. Prior studies used inclusion criteria (such as inclusion by fetal growth restriction) that is not endorsed by ACOG for defining severe versus non-severe preeclampsia [17], varying criteria for defining elevated ONSD in preeclampsia cohorts [15], or lacked cohorts to assess differences by neurologic criteria [13].

As preeclampsia has similar imaging findings as hypertensive encephalopathy, utilizing ONSD as a marker of severity for preeclampsia as for hypertensive encephalopathy is biologically plausible. The physiology of raised intracranial pressure with vasogenic edema from preeclampsia would logically apply to the use of ocular ultrasound as an objective measure of severity. All images were obtained by a single sonographer, removing inter-observer variability.

The limitations of this research include being a single site study. Additionally, a cut-off value for ONSD, such as above 5.8 mm, has not been validated in an obstetric cohort to predict increased ICP. Assessing ONSD prior to the initiation of antihypertensive medications and magnesium or assessing pre- and post-magnesium values would control for a common intervention which could have impacted measurements. It has been found in prior studies on non-pregnant adults that there are rapid changes in ONSD with changing cerebrospinal fluid pressure [29]. However, prior studies have found that CPP [30] can be pathologically elevated even after treatment of elevated blood pressure for several days [15], which would make future endeavors consider timing of treatment as a variable, but not a contraindication to obtaining an ONSD measurement. Additionally, there is a lack of comparison to direct ICP measurements, or indirect measurements with magnetic resonance imaging [26].

Unfortunately, as we enrolled by convenience sampling, self-selection bias is a possible confounder, and chronic hypertension was not controlled for during enrollment. It is known that the background rate of intracranial hypertension is higher in women with chronic hypertension and obesity [31]. Although BMI was not found to be significantly different between groups, the incidence of chronic hypertension was variable between the cohorts.

The use of the linear probe may not be available in all labor and delivery units, and evaluation with use of the more common curvilinear probe would be of interest. However, studies comparing measurements made in the visual axis versus the coronal axis have shown significant differences when using a curvilinear probe [32] and decreased variability in ONSD with different axis of measurement found when using a linear transducer [33]. There are limited studies assessing standard error of optic nerve sheath ultrasound images, as the acoustic shadow for any optic nerve sheath would be expected to have a consistent error for any measured ONSD with the same probe. However, the error has been assumed to be less than 0.1 to 0.2 mm by ONSD models utilizing Sonosite L25 linear probes [34].

Obtaining larger sample sizes would improve the ability to control for potential confounders, such as timing of antihypertensive medications or use of magnesium, chronic hypertension [31], class III obesity, age [8], or to assess for racial variations, which have been found to be risk factors for the development of preeclampsia itself [35, 36]. A powered sample size for each group would also improve the sensitivity of finding a difference in ONSD magnitude between severe preeclampsia, preeclampsia without severe features, and control cohorts, if there is indeed one between the groups. Finally, obtaining baseline measurements of ONSD in pregnant women in all three trimesters would allow for improved interpretation of ONSD in future studies.

## Conclusion

Optic nerve sheath diameters were not found to be significantly different between severe preeclampsia patients with neurologic features and healthy control patients, nor was there a significant difference between any of the cohorts’ ONSD means.

This study does not support that ONSD is associated with hypertensive disease severity. Continuing to search for an objective measure to differentiate severe preeclampsia from a chronic hypertension exacerbation would be clinically advantageous, as several subjective symptoms such as headache, visual changes, or right upper quadrant pain can be benign findings in pregnancy. Hypertension in pregnancy is acknowledged to carry a spectrum of risk, and early delivery is indicated for many women. However, identifying an objective measure of risk could be useful in stratifying the need for early term delivery to avoid complications of premature delivery and for maternal management.

## Data Availability

Datasets used and analyzed for the current study are available from the corresponding author on reasonable request, and all data analyzed during this study are included in this article.

## Abbreviations

ONSD: Optic Nerve Sheath Diameter
ACOG: American College of Obstetrics and Gynecology
PE: Preeclampsia
sPE: Severe Preeclampsia
MUSC: Medical University of South Carolina
PRES: Posterior reversible encephalopathy syndrome
HTN: Hypertension
DM: Diabetes mellitus
